# Immunoproteomic Screening of Candidate Antigens for the Preliminary Development of a Novel Multi-Component and Multi-Epitope Vaccine Against *Streptococcus suis* Infection

**DOI:** 10.3390/vaccines13101020

**Published:** 2025-09-30

**Authors:** Yue Zhang, Caiying Li, Yutong Feng, Qibing Gu, Jinwang Hu, Yuhang Li, Lu Xia, Shaopo Zu

**Affiliations:** 1 Ministry of Education Key Laboratory for Animal Pathogens and Biosafety, College of Veterinary Medicine, Henan Agricultural University, Zhengzhou 450046, China; yueruda@163.com (Y.Z.); lcy2021255618@163.com (C.L.); feng2tong1023@163.com (Y.F.); m17329270701@163.com (J.H.); liyuhang202507@163.com (Y.L.); xialulwj201@163.com (L.X.); 2 College of Veterinary Medicine, Nanjing Agricultural University, Nanjing 210095, China; 2020207030@stu.njau.edu.cn; 3 Henan Province Key Laboratory for Animal Food Pathogens Surveillance, Zhengzhou 450046, China

**Keywords:** *Streptococcus suis*, immunoproteomics, protective antigen, multi-component subunit vaccine, multi-epitope vaccine

## Abstract

**Background/Objectives:** *Streptococcus suis* (SS), an important zoonotic pathogen, has caused significant economic losses to the global pig industry. Existing commercial vaccines for SS mainly provide effective protection against a single serotype. Due to the existence of many serotypes and their robust immune evasion capabilities, the development of multi-component subunit vaccines or multi-epitope vaccines that provide effective cross-protection against different strains of SS is a key focus of current research. **Methods:** We applied two-dimensional electrophoresis (2-DE) and immunoblotting to screen for candidate immunogens among the immunogenic cell wall proteins of SS. BALB/c mice were immunized intradermally with a multi-component, multi-epitope vaccine. The vaccine’s safety and immunogenicity were assessed via clinical monitoring, antibody titer detection, cytokine assays, and survival curve analyses. **Results:** In this study, eight immunogenic cell wall proteins (GH25, Pk, PdhA, Ldh, ExoA, Pgk, MalX, and Dnak) were successfully identified using MALDI-TOF-MS, all of which could induce high IgG antibody titers. Based on the conservation and immunoprotection demonstrated by these eight protective antigenic proteins, PdhA, Ldh, and MalX were screened to construct a multi-component subunit vaccine as a candidate vaccine for providing cross-protection against SS isolates of multiple serotypes. Challenge studies showed that mice immunized with the multi-component subunit vaccine (PdhA, Ldh, and MalX) were protected against challenges with the SS2 virulent strain ZY05719 (62.5% protection) and the SSChz virulent strain CZ130302 (75% protection). Subsequently, we utilized immunoinformatics techniques to design a novel multi-epitope vaccine (MVPLM) derived from the immunogenic proteins PdhA, Ldh, and MalX. However, challenge tests revealed that the MVPLM offered limited protection against SS. **Conclusions:** These data demonstrate that a multi-component subunit vaccine composed of PdhA, Ldh, and MalX proteins shows promise as a candidate universal vaccine against multiple SS serotypes.

## 1. Introduction

*Streptococcus suis* (SS) is an important zoonotic pathogen that causes sepsis, pneumonia, endocarditis, arthritis, and meningitis, and is widely distributed worldwide, especially in Asia and Europe [1,2]. The total infection rate of SS in pig farms in China, France, and Thailand ranges from 41.0% to 72.0%, resulting in huge economic losses to the pig industry [3,4,5]. At present, the main method for controlling SS in livestock production is the use of antimicrobials. However, the frequent emergence of drug-resistant strains of SS and the transfer of resistance genes posed a significant risk to public health [6,7]. The development of SS vaccines is important for reducing reliance on antibiotics and for safeguarding public health.

SS isolates are serologically classified into 29 classical serotypes (1–19, 21, 23–25, 27–31, and 1/2) and 28 new novel cps loci (NCL) serotypes (NCL1-NCL26, NCL-21, and Chz) [8], according to the antigenicity of capsular polysaccharide (CPS). SS can also be divided into 2910 ST types [4,9] based on its sequence of 7 home guarding genes (*recA*, *cpn60*, *gki*, *dpr*, *mutS*, *aroA*, and *thrA*). Due to this large number of SS serotypes and ST types, the SS epidemic in China appears to be in a dynamic stage of evolution, and the conventional single-inactivated vaccine can no longer meet market demands [1]. CPS is an important virulence factor of SS that plays a key role in its pathogenesis [1,8,10]. However, CPS is a poor immunogen in SS; as a non-thymus-dependent antigen, it cannot induce a sufficient immune and recall response [11]. Studies have shown that SS2 CPS conjugated with tetanus toxoid induces T-cell-dependent responses, and the induced IgM and IgG protect mice and pigs from attack by homologous bacteria [12,13]. SS comprises numerous serotypes, and a single CPS can generally protect against different strains of the same serotype. This makes it difficult to provide good cross-immune protection, especially against strains with capsular defects. Certain virulence factors, such as hemolysin (SLY), muramidase-released protein (MRP), and extracellular factor (EF) have been shown to protect animals against homologous or heterologous strains [14,15,16]. Although SLY, MRP, and EF are virulence factors of SS2, they are not conserved nor widespread in other serotypes of SS [17,18]. Therefore, it is necessary to screen for more conserved immunogenic antigens from different serotypes as candidate vaccines.

Many SS surface proteins are involved in the pathogenesis of bacteria and have been shown to induce strong immune responses. For example, Sao protein is a common surface protein that is highly conserved in SS strains. Li et al. confirmed that immunization with recombinant Sao protein can confer strong humoral immunity and cross-protection against various SS serotypes in mouse and pig animal models [12,19]. Hsueh et al. immunized pregnant sows with recombinant Sao protein plus inactivated SS vaccine, which provided their piglets with passive immunity and protection against attack from homologous and heterologous SS strains [20]. Ide*_Ssuis_* is a conserved virulence factor of SS that has been shown to induce higher antibody levels and provide immune protection to piglets [21,22]. In addition, several other protective antigens have been identified, including ornithine carbamoyltransferase (OCT), phosphate ABC transporter ATP-binding protein (PstB), and peptidyl isomerase (PrsA) [23,24,25]. Multi-component subunit vaccines or multi-epitope vaccines offer greater protective efficacy in livestock vaccination strategies as compared with single antigen vaccines. The vaccine candidate rSC0016—which combines SS2-Sao and SS9-Enolase—has recently been shown to provide favorable cross-protective effects against SS2, SS7, SS9, and SS 1/2 in mice models [26]. Using immunoinformatics, Liang et al. designed a multi-epitope vaccine (MVSS) that effectively reduced histopathological damage in mice caused by SS2 and SSChz strains [27]. Presently, it remains essential to broaden the data on the protective effects of multi-component subunit vaccines or multi-epitope vaccines against SS.

Immunoproteomics combines proteomics and serological analysis to improve the identification of proteins recognized by antibodies in immune sera. This enables the effective evaluation of antigens as vaccine candidates [28]. Hu et al. used this technique to identify whole-cell bacterial proteins of *Riemerella anatipestifer.* They successfully identified 34 ideal immunogenic proteins to reveal the bacteria’s pathogenesis and develop new candidate vaccines [29]. Faria et al. applied immunoproteomics to search for immunogenic proteins involved in the serum diagnosis of *brucellosis*. They successfully identified three key proteins, namely, MDH (malate dehydrogenase), SOD (superoxide dismutase), and multispecies sugar ABC transporter [30].

**Figure 1:**
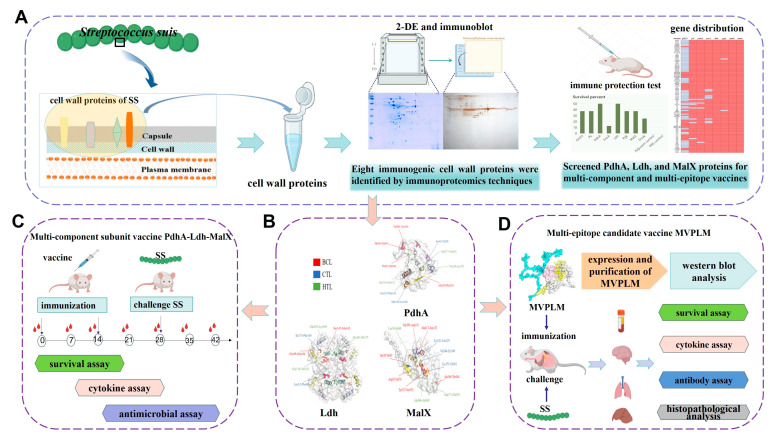
The workflow that screen candidate antigens by immunoproteomics to develop novel multi-component and multi-epitope vaccines against SS infection. (**A**) Eight candidate cell wall immunogens from the SS were isolated using immunoproteomics. (**B**) The three-dimensional structures and anticipated epitopes of the PdhA, Ldh, and MalX proteins. (**C**,**D**) Assessment of multi-component and multi-epitope vaccines immune protective effects. The figure was created using BioRender (https://www.biorender.com/) (1 September 2025).

In this study, we used immunoproteomics to screen for immunoprotective antigens of SS. These antigens were expressed in vitro and challenged for immune protection. The multi-component subunit vaccine and multi-epitope vaccine developed according to the conservation and immunoprotective effects of eight candidate antigen proteins (based on the PdhA, Ldh, and MalX proteins) provided positive cross-protection against different strains of SS. The workflow was shown in Figure 1. These results represent a new avenue of research into the development of subunit SS vaccines.

## 2. Materials and Methods

### 2.1. Bacterial Strains and Culture Conditions

The strain CZ130302 (as the representative strain of the SS serotype Chz) was isolated from a meningitis outbreak with a high mortality rate in piglets. The SS2 strain ZY05719 was isolated from a pig with septicemia. The SS strains were cultured either in Todd Hewitt broth (THB; Becton-Dickinson, Franklin Lakes, NJ, USA) containing 5% fetal bovine serum or in solid medium containing 5% (*v*/*v*) sheep blood, at 37 °C in a 5% CO_2_ atmosphere.

### 2.2. Cell Wall Protein Extraction

The cell wall proteins were extracted according to previously reported methods [31]. The CZ130302 strain was cultured to its exponential phase, centrifuged at 13,800× *g* for 10 min at 25 °C using a Gallop DH-18K centrifuge (GallopTech, Shanghai, China) with the DH18K-A0224 rotor (GallopTech, Shanghai, China), and washed three times with sterile PBS. The bacteria were resuspended and dissolved in 4 mL preparation solution (125 U/mL Mutanolysin, 25% sucrose, 30 mM Tris-HCl, and 3 mM MgCl_2_), and incubated for 1.5 h. The supernatant was then collected via centrifugation at 13,800× *g* for 10 min at 25 °C, and filtered twice through a 0.22 μm filter. Trichloroacetic acid (TCA) was then added with a final concentration of 10% before incubating the sample on ice for 30 min. Thereafter, the precipitate was collected via centrifugation, washed twice with cold acetone to remove TCA, air-dried at room temperature to a semi-air-dried state, and stored at −20 °C for later use.

### 2.3. Immunogenic Proteins Identified by 2D-Western Blot

The extracted 200 µg cell wall protein samples were dissolved in 250 µL preparation solution at room temperature for 30 min, then centrifuged at 13,800× *g* for 20 min at 25 °C. Two equivalent protein samples were treated with a 2-D Clean-up Kit (GE Healthcare, Chicago, IL, USA) and resuspended in 250 µL hydration solution (2 M thiourea, 7 M urea, 0.5% *v*/*v* IPG buffer, 0.2% *w*/*v* DTT, 2% *w*/*v* CHAPS, and 0.002% *w*/*v* bromophenol blue). Subsequently, two equal portions were added to the strip (Immobiline DryStrip, pH 4–7; GE Healthcare, Chicago, IL, USA), and hydrated at room temperature for 12 h (30 V). Isoelectric focusing (IEF) was applied for a total of 11.5 h: 500 V (4 h), 1000 V (1 h), 2000 V (1 h), 4000 V (1 h), and 8000 V (4.5 h).

The cell wall proteins were separated using 12% acrylamide electrophoresis and then transferred onto PVDF membranes (Millipore, WWLP, Boston, MA, USA). The membranes were blocked with Tris-buffered Saline Tween (TBST) containing 5% (*w*/*v*) skim milk for 1.5 h at room temperature, and then incubated with CZ130302 hyperimmune serum (diluted 1:1000) at 37 °C for 2 h. Unincubated serum membranes were used as a blank control group. After washing three times with TBST, rabbit anti-pig antibody (diluted 1:5000) was added and incubated at 37 °C for 1 h. Subsequently, the processed membranes were stained with substrate solution 3,3′-diaminobenzidine (Tiangen, Beijing, China).

### 2.4. MALDI-TOF-MS and Database Retrieval

Using ImageMaster 7.0, immunoreactive protein spots on the PVDF membrane were identified on the 2D gels, which were then analyzed using matrix-assisted laser desorption ionization-time of flight mass spectrometry (MALDI-TOF-MS) (Shanghai Applied Protein Technology Co., Ltd. Shanghai, China). Peptide mass fingerprinting (PMF) data were obtained using MASCOT (http://www.matrixscience.com) (6 January 2019). To validate the protein spots, the MALDI-TOF data match had to comprise > 4 peptides with a simultaneous sequence coverage of >15%. Proteins with a MASCOT score that exceeded the threshold were considered reliable, while the proteins with a lower MASCOT score were either rejected or verified manually.

### 2.5. Cloning, Expression, and Purification of the Recombinant Proteins

Once the identified proteins were analyzed, their corresponding sequences were identified in the CZ130302 strain (CP024974.1) via BLASTp searching. A total of eight amplified target fragments were inserted into the pET28a vector using EcoRI/BamHI and XhoI restriction sites and transformed into *E. coli* BL21 (DE3). Purification and ultrafiltration of the recombinant proteins were achieved using HisTrap™ HP (GE Healthcare, Chicago, IL, USA) and membrane ultrafiltration (Millipore, 10,000 NMWL, WWLP, Boston, MA, USA), respectively. The quality of the purified proteins was analyzed using SDS-PAGE and Western blot. Finally, the concentration of the recombinant proteins was measured using a BCA Protein Assay (Thermo, Waltham, MA, USA) and stored at −80 °C. All the primers used in this study are listed in Table S1.

### 2.6. Multi-Epitope Vaccine Designing and Processing

In this study, ABCpred was employed to predict the B-cell (BCL) epitopes for PdhA, Ldh, and MalX proteins [32]. Epitopes exhibiting an antigenicity threshold > 0.85 were selected for further analysis. The epitopes’ allergenicity and toxicity were measured using Allertop v.2 and Toxin Pred 2 server (https://webs.iiitd.edu.in/raghava/toxinpred2/) (31 October 2024), respectively. Additionally, the Immune Epitope Database (IEDB) was used to predict the cytotoxic T lymphocyte (CTL) epitopes, and the helper T lymphocyte (HTL) epitopes were predicted using the IEDB MHC-II tool (https://www.iedb.org/) (2 November 2024) [33,34]. The epitopes identified in the preceding phase were assessed and filtered according to their antigenic properties and capabilities to induce cytokines using IL4pred. The candidate epitopes were evaluated based on their antigenic, immunogenic, and toxicity profiles. To minimize junctional immunogenicity, promiscuous epitopes were conjugated with an appropriate adjuvant, namely, the 50S ribosomal L7/L12 protein. Suitable linkers (such as AAY, KK, and GPGPG) were also incorporated. Molecular docking analyses were performed to validate the vaccine’s structural integrity in conjunction with the TLR-4 complex [35]. To facilitate the effective expression of multi-epitope vaccine protein within the prokaryotic expression system (specifically *E. coli* BL21 (DE3)), we used JCat to perform codon optimization of the amino acid sequence, thereby generating an enhanced DNA sequence. The vaccine’s optimized DNA sequence was then conjugated with the pET28a (+)-sumo vector, and the MVPLM protein was finally expressed.

### 2.7. Immunization and Challenge Studies

In this study, five-week-old female SPF BALB/c mice were used as immunoprotection subjects [27,36]. All the animal experiments were performed in strict accordance with the Animal Research Committee Guidelines of Jiangsu Province (License Number: SYXK(SU)2021-0086). The purified recombinant proteins were emulsified with ISA 201 adjuvant (SEPPIC, Paris, France) at a ratio of 1:1. Mice were injected intradermally with purified proteins at a dose of 50 µg/mouse. Mice injected with PBS emulsified in ISA 201 adjuvant were used as negative controls, and mice injected with PBS alone were used as blank controls. Furthermore, the commercialized inactivated vaccine (SS2 HA9801 strain; 2021 New Veterinary Drug Certificate No. 29) was also used as a control group in the experiment. The vaccines were initially injected at day 0 and boosted at day 14. Meanwhile, we collected serum samples from the mice before immunization and again at 7, 14, 21, 28, 35, 42, and 70 days after immunization. To reduce pain and stress responses, the mice were first anesthetized with isoflurane, and blood samples were collected from the ophthalmic vein to separate serum. No more than 150 µL of blood was collected per mouse per session. Additionally, bleeding was minimized by rapid cotton-ball pressing immediately after blood collection. For the challenge test, 2 × 10^7^ CFU/mouse of CZ130302 or 2 × 10^8^ CFU/mouse of ZY05719 were injected intraperitoneally into the mice in each group (*n* = 16). To confirm the vaccine’s safety, the relative percentage survival was recorded at least two weeks days after the challenge. After the experiment, the mice were first anesthetized with isoflurane; then, rapid cervical dislocation was performed in accordance with ethical and technical guidelines to ensure euthanasia.

### 2.8. Histopathological Analysis

The brains, lungs, and livers of the mice were preserved using a 4% paraformaldehyde solution, and subsequently embedded in paraffin. The samples were sectioned into 5 µm thick slices, mounted onto glass slides, and stained with hematoxylin-eosin (H&E) to be examined under a light microscope.

### 2.9. Antibody Determination

To measure the specific IgG antibodies, all titers of the collected serum were determined via indirect ELISA. 96-well microtiter plates were first coated with purified recombinant proteins (0.5 μg/well) and incubated overnight at 4 °C. Then, the Ag-coated plates were blocked with 0.5% BSA at 37 °C for 2 h to prevent nonspecific immune reactions. Serially diluted antiserums collected from the mice were added and incubated at 37 °C for 1 h. Next, the binding of the antibodies to the antigens was detected using horseradish peroxidase (HRP) conjugated goat-anti-mouse IgG (Thermo, Waltham, MA, USA), diluted at a ratio of 1:5000. The color reaction was developed using 100 μL of 3,3′,5,5′-tetramethylbenzidine (TMB) substrate solution. Each plate was read using a microplate reader (Bio-Tek Instruments, Winooski, VT, USA) at 450 nm.

### 2.10. Adherence Inhibition Assays

To evaluate the inhibitory effect of the antibodies on bacterial adherence, adherence inhibition assays were performed according to the method described by Pan [37]. 24-well plates were first coated with 1% BSA overnight at 4 °C. HEp-2 and HBMEC cells were cultured into monolayer cells (~5 × 10^5^ cells/well) in the treated plates. In addition, the CZ130302 strain (1 × 10^7^ CFU/mL) was incubated with the antiserum (1:20 dilution) at 37 °C for 30 min, and co-incubated with negative serum as a negative control. The pretreated bacteria were then added to wells containing HEp-2 and HBMEC cells, centrifuged at 14× *g* for 15 min using a Thermo Scientific Multifuge X4R Pro centrifuge (Thermo, Waltham, MA, USA) with the M20 Microplate rotor (Thermo, Waltham, MA, USA), and left to stand at 37 °C for 2 h. After washing five times with sterile PBS, the cells were lysed with 100 μL 0.25% trypsin-EDTA and 900 μL sterile deionized water, followed by agar plating and bacterial enumeration.

### 2.11. Distribution Analysis

To detect the prevalence and gene distribution of the eight candidate proteins, 92 clinical strains of SS serotypes 2, 3, 7, 9, and Chz were isolated from different regions across China. The detection primers for the target genes are listed in Table S1. In addition, the gene distributions of the 92 strains of SS (as described previously and determined via PCR analysis) are listed in Table S2.

### 2.12. Indirect Immunofluorescence Analysis

Immunofluorescence microscopy assays were performed to visualize the localization of the eight candidate proteins at the SS surface. CZ130302 and ZY05719 were cultured to an OD_600_ of 0.4–0.6 before the bacteria were collected via centrifugation at 2400× *g* for 10 min at 25 °C. After washing twice with PBS, the bacteria were resuspended in 2 mL PBS. The 5 µL bacterial solution was evenly coated onto the cover glasses. After drying, 500 µL 4% paraformaldehyde was added and fixed for 15 min. The slides were then blocked with 5% BSA and disposed with hyperimmune serum (diluted 1:1000) at 37 °C for 2 h. The blank control group was incubated with pre-immune serum. After washing three times with PBST, sheep anti-mouse FITC fluorescent antibody (diluted 1:1000) was added and incubated at 37 °C for 1 h. Additionally, the bacterial DNA was stained with a mounting medium containing DAPI. The treated samples were finally visualized using a laser scanning confocal microscope (Zeiss LSM 800, Baden-Württemberg, Germany).

### 2.13. Antimicrobial Assay In Vitro

Antimicrobial assays were conducted in vitro to evaluate the antimicrobial efficacy of the multi-component subunit vaccine antibodies against various serotypes of SS strains. The activated SS strains were cultured to an OD_600_ of 0.4–0.6 and diluted 50-fold with THB solution. The immune serums of the vaccine, the PBS control, and the adjuvant control groups were also diluted 50-fold in THB. The SS strains were incubated with immune serum at a ratio of 1:1 at 37 °C for 4 h. The colony-forming units were enumerated the following day. The PBS serum group served as the control baseline, and the experiments were repeated four times.

### 2.14. Statistical Analysis

GraphPad Prism version 10 was used to analyze and draw the data. The Mantel–Cox log-rank test was used to analyze the survival rate of the mice. The Shapiro–Wilk test was used to assess the normality of the data distribution. The homogeneity of variances was assessed using Brown-Forsythe test. The statistical significance of the MVPLM-specific antibody was assessed using two-way analysis of variance, followed by Tukey’s multiple comparison test. The statistical significance of the adherence inhibition, antimicrobial, and cytokine assays was assessed by one-way analysis of variance. Significant findings were further evaluated with post hoc tests: Tukey’s test was used for all pairwise comparisons, while Dunnett’s test was used for comparisons against a single control group. A post hoc power analysis was performed for our key findings using G*Power 3.1.9.7 software. Data were expressed as mean ± standard error of the mean (mean ± SEM), and differences were indicated when *p*-values < 0.05 (ns *p* ≥ 0.05, * *p* < 0.05, ** *p* < 0.01, *** *p* < 0.001, **** *p* < 0.001).

## 3. Results

### 3.1. Eight Candidate Immunogens Identified by Proteomics and 2D Western Blot Assays

**Figure 2:**
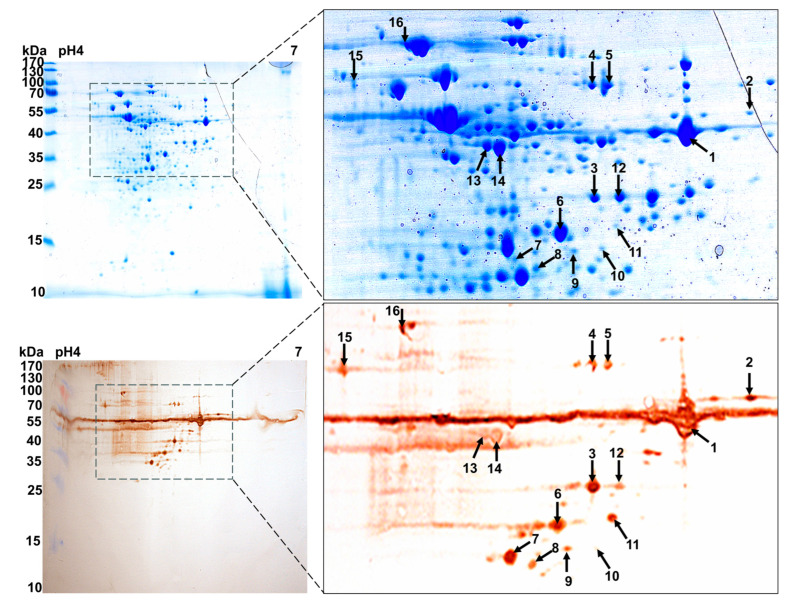
Two-dimensional electrophoresis and Western blot analysis of strain CZ130302. The 2D separation profile for the cell wall proteins of the SS virulent strain CZ130302, stained with Coomassie brilliant blue G250. Another gel was transferred to a PVDF membrane and reacted with CZ130302 high-immune serum from pigs. The immunoreactive spots were marked in their corresponding positions with numbers (1–16).

Bacterial wall proteins are considered good vaccine candidates due to their susceptibility to antibodies [38,39]. In the field of bacterial vaccine research, immunoproteomics has been used to identify pathogen-associated antigens as vaccine candidates. To identify more immunoreactive proteins, the cell wall proteins of the SS hypervirulent strain CZ130302 were isolated using 2D SDS-PAGE and analyzed by Western blot. As shown in Figure 2, more than 200 G250 Coomassie brilliant blue-stained protein spots were identified on the 2-DE gel, with a molecular weight of 15–110 kDa. Using ImageMaster 7.0, the corresponding immunoreactive protein spots on the PVDF membrane were observed on the Coomassie brilliant blue staining glue. Finally, a total of eight immunoreactive proteins were successfully identified using MALDI-TOF-MS, and the NCBI sequence database was used to match them. The theoretical molecular weights, the number of matching peptides, and the protein scores are summarized in Table 1.

**Figure 3:**
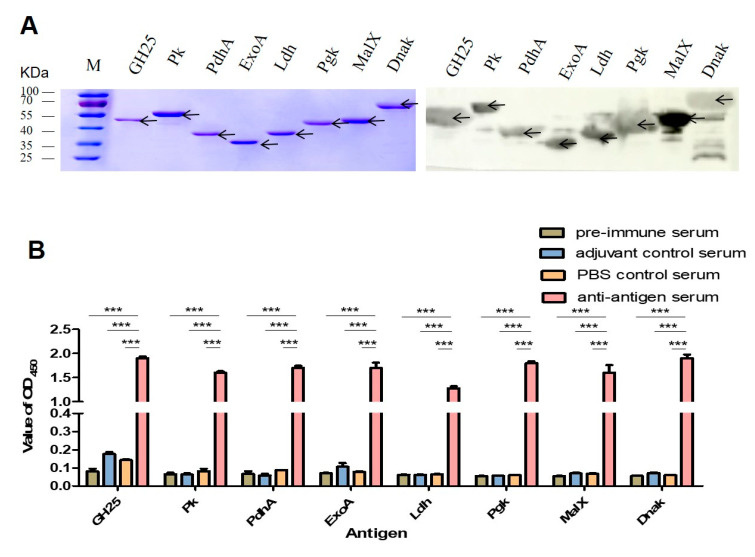
Immune protein expression, purification, and immunization. (**A**) SDS-PAGE and Western blot analysis of the eight recombination proteins. Arrows indicate the specific bands corresponding to the target proteins in the SDS-PAGE and immunoblot assays. (**B**) Titers of IgG antibodies to the individual subunit proteins among vaccinated mice (*n* = 3). Error bars represent the standard error of the mean (SEM). All samples were analyzed using one-way analysis of variance, followed by Tukey’s multiple comparison test (*** *p* < 0.001 for comparisons between anti-antigen serum and both pre-immune and adjuvant/PBS control groups). The adequacy of the sample size is supported by post hoc power analysis (lowest Cohen’s d = 8.14).

The expression vector pET-28a was used in vitro to determine the immunoreactivity of the eight identified proteins. The SDS-PAGE profile (Figure 3A) shows that all eight recombinant proteins were successfully expressed following 1 mM IPTG induction and metal affinity (Ni-NTA) chromatography purification. The Western blot results confirm that the recombinant proteins GH25, Pk, PdhA, Ldh, ExoA, Pgk, MalX, and Dnak could react with the hyperimmune serum.

**Figure 4:**
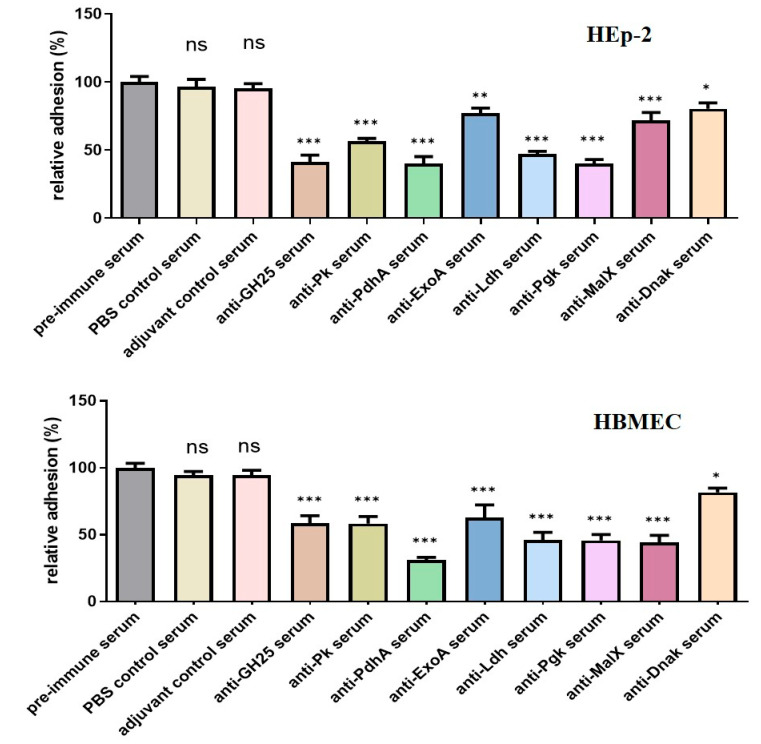
The inhibitory effect of the recombinant protein antiserum on the adhesion ability of CZ130302 to HEp-2 and HBMEC cells. CZ130302 incubated with pre-immune serum served as the blank control. The contribution of the antiserum to the adherence of bacteria to the HEp-2 (**above**) and HBMEC (**below**) cells. Mean ± SEM of three independent experiments, each performed in triplicate. All samples were analyzed using one-way analysis of variance, followed by Dunnett’s multiple comparison test (* *p* < 0.05; ** *p* < 0.01; *** *p* < 0.001, compared to pre-immune serum group). The large observed effect sizes (Cohen’s d > 2.70 for all protein antiserum vs. pre-immune serum comparisons) resulted in the statistical power greater than 90% (α = 0.05).

Due to the importance of vaccine safety and immunization potency, the safety of eight recombinant subunit vaccines was evaluated by observing the mental state, growth, and mortality of the vaccinated animals. No animals died during the entire process of immunization, and the mice in the experimental group and control group remained symptom-free. Serum was collected 14 days after the second vaccination. The immunized mice’s serum IgG levels were measured via indirect ELISA to evaluate the humoral response to the recombinant proteins’ antigen specificity. Compared with the pre-immune serum levels and adjuvant/PBS controls, the immunogen-specific IgG antibody levels were significantly increased in nearly all the recombinant protein immunized groups (Figure 3B). Adhesion inhibition assays were performed to evaluate the antibodies’ protective efficacy against bacterial adherence. As shown in Figure 4, the SS strain CZ130302 incubated with the recombinant protein antiserum exhibited significantly reduced adhesion ability to HEp-2 and HBMEC cells as compared with the control groups.

### 3.2. The Protective Efficacy of Eight Vaccine Candidates In Vivo

Following two immunizations with the recombinant proteins, the mice underwent a challenge protection test. Compared with the control group, the survival rate of the mice immunized with the vaccine candidates was greatly improved (Table 2). In the groups challenged with SS CZ130302, the PdhA and Ldh immune groups demonstrated a relatively high rate of protection (50%), followed by the GH25, Pk, Pgk, and MalX immune groups, each of which showed a protective effect of 37.5%. The ExoA and Dnak proteins had a weak protective effect with only 12.5% and 25%, respectively.

### 3.3. Alignment and Distribution of the Eight Vaccine Candidates

**Figure 5:**
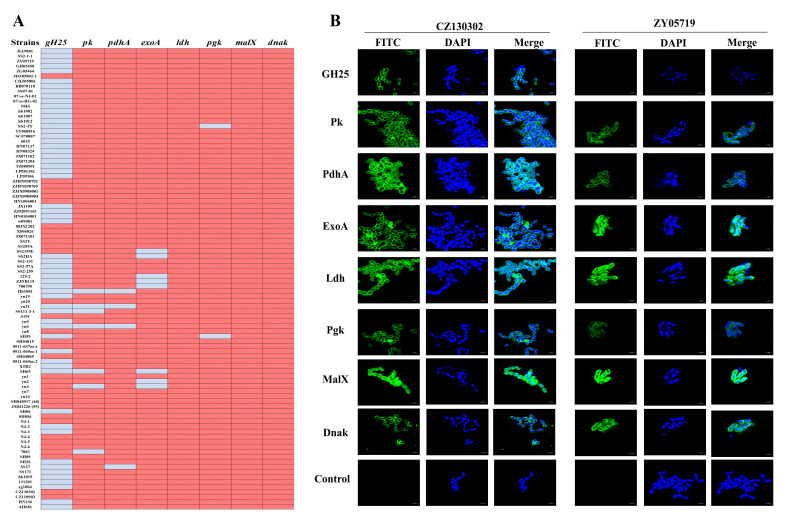
The conservation of eight candidate proteins in *Streptococcus suis*. (**A**) Gene distribution of eight candidate proteins in *Streptococcus suis* serotypes 2, 3, 7, 9, and Chz. (**B**) Expression of eight candidate proteins in the SSChz virulent strain CZ130302 and the SS2 virulent strain ZY05719.

Since there are currently at least 57 known serotypes of SS, the conservative identification of protective antigens is essential for the development of effective vaccines. The results of NCBI comparative analysis showed that the *pk*, *pdhA*, *exoA*, *ldh*, *pgk*, *malX*, and *dnak* genes were highly conserved. The sequence homology in SS was >90%, while that of the *gH25* gene was relatively poor at only 70%. We used 92 clinical isolates of SS serotypes 2, 3, 7, 9, and Chz preserved in our laboratory to detect the gene distributions of the eight vaccine candidates. The conservative detection primers were designed according to the gene sequence published in the NCBI and the measurements conducted in our laboratory. As shown in Figure 5A and Table S2, *ldh*, *malX*, and *dnak* were all detected in the 92 strains. The detection rates of *pk*, *pdhA*, *exoA*, and *pgk* were 92%, 96%, 91% and 98%, respectively. Among these, the detection rates of *pk* and *pdhA* in SS2 were both 100%. In addition, the target fragment of *gH25* was detected in only 34 strains. The indirect immunofluorescence results also showed that all the proteins except for GH25 were well expressed in the SS2 virulent strain ZY05719 (Figure 5B).

### 3.4. Improved Immune Protection of the Multi-Component Subunit Vaccine Composed of PdhA, Ldh, and MalX Proteins

**Figure 6:**
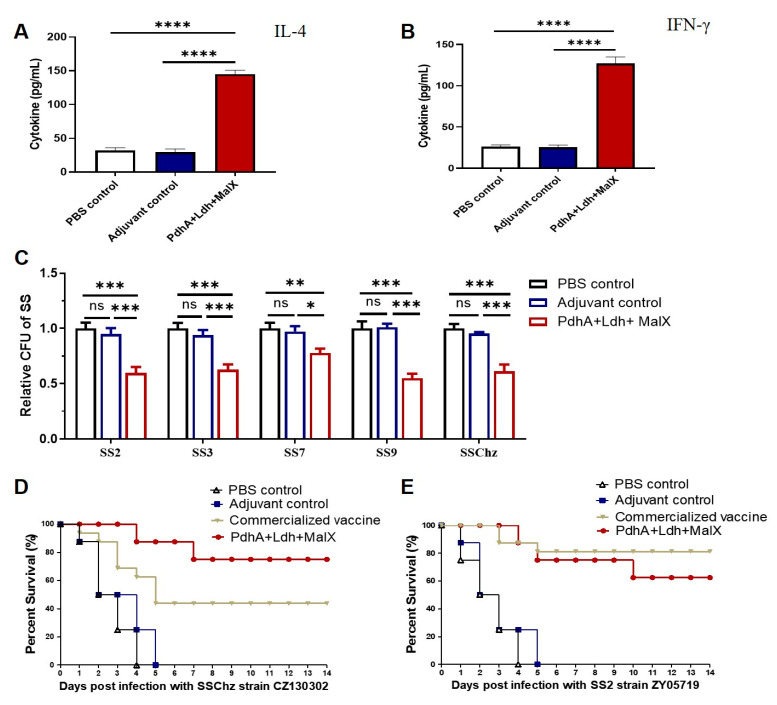
Cytokine levels and survival rates of mice vaccinated with the PdhA-Ldh-MalX multi-component subunit vaccine. (**A**,**B**) Serum was collected from twice-immunized mice and analyzed to determine their IL-4 (**A**) and IFN-γ (**B**) cytokine levels via ELISA. Serum samples taken from mice immunized with the multi-component subunit vaccine exhibited significantly higher levels of IL-4 and IFN-γ compared with the PBS/adjuvant controls. The data represents a mean of 6 biological replicates ± SEM. All samples were analyzed using one-way analysis of variance, followed by Tukey’s multiple comparison test (**** *p* < 0.0001). (**C**) Antimicrobial activity test of the PdhA-Ldh-MalX multi-component subunit vaccine’s polyclonal antibodies. The immune serum markedly suppressed the growth of multiple SS serotypes. The results are indicated as the means ± SEM from 4 independent experiments. All samples were analyzed using one-way analysis of variance, followed by Tukey’s multiple comparison test (* *p* < 0.05; ** *p* < 0.01; *** *p* < 0.001). The values of Cohen’s d for the vaccine group versus both control groups exceeded 2.0 (with statistical power (1 − β) > 0.91). (**D**,**E**) The immune protective effect of the PdhA-Ldh-MalX multi-component subunit vaccine on mice challenged with SS CZ130302 (**D**) and ZY05719 (**E**) (*n* = 16 per group).

Our results showed that the protection provided by a single subunit vaccine may be limited. According to the conservation and immune protection of the eight protective antigen candidate proteins, the PdhA, Ldh, and MalX proteins were screened to develop a multi-component subunit vaccine. The serum of immunized mice was collected 14 days after the administration of the second vaccination. The induction levels of IFN-γ and IL-4 cytokines in these samples were analyzed via ELISA. IFN-γ and IL-4 could serve as indicator cytokines for Th1-type and Th2-type immune responses in SS infection. The serum samples of mice immunized with the multi-component subunit vaccine produced significantly higher levels of both IFN-γ and IL-4 compared with the PBS/adjuvant controls (Figure 6A,B). The antibody inhibition assay is a straightforward and efficient method for evaluating the antimicrobial efficacy of the multi-component subunit vaccine antibody against various SS serotypes. The immune serum was incubated for 3 h with the five serotypes of SS (SS2, SS3, SS7, SS9, and SSChz) before bacterial colony counts were performed. The results showed that the PdhA-Ldh-MalX multi-component subunit vaccine antibody significantly inhibited the growth of these SS serotype strains (Figure 6C).

The mice immunized with the PdhA-Ldh-MalX multi-component subunit vaccine showed an immunoprotective efficiency of up to 75% against SS CZ130302, whereas the mice in the adjuvant/PBS control group all died within 5 days (Figure 6D). In addition, the multi-component subunit vaccine effectively protected the mice against infection from the SS2 virulent strain ZY05719, providing 62.5% immune protection (Figure 6E). Furthermore, the commercialized inactivated vaccine (SS2 HA9801 strain), which is widely used clinically, provided a high protective efficacy of 81.25% against SS ZY05719 but exhibited a weaker protective efficacy of 43.75% against the highly virulent Chz strain CZ130302. These data indicate that the PdhA-Ldh-MalX multi-component vaccine demonstrates a slight advantage in resisting different SS serotypes.

### 3.5. Development of a Multi-Epitope Candidate Vaccine (MVPLM) Using Immunoinformatics

**Figure 7:**
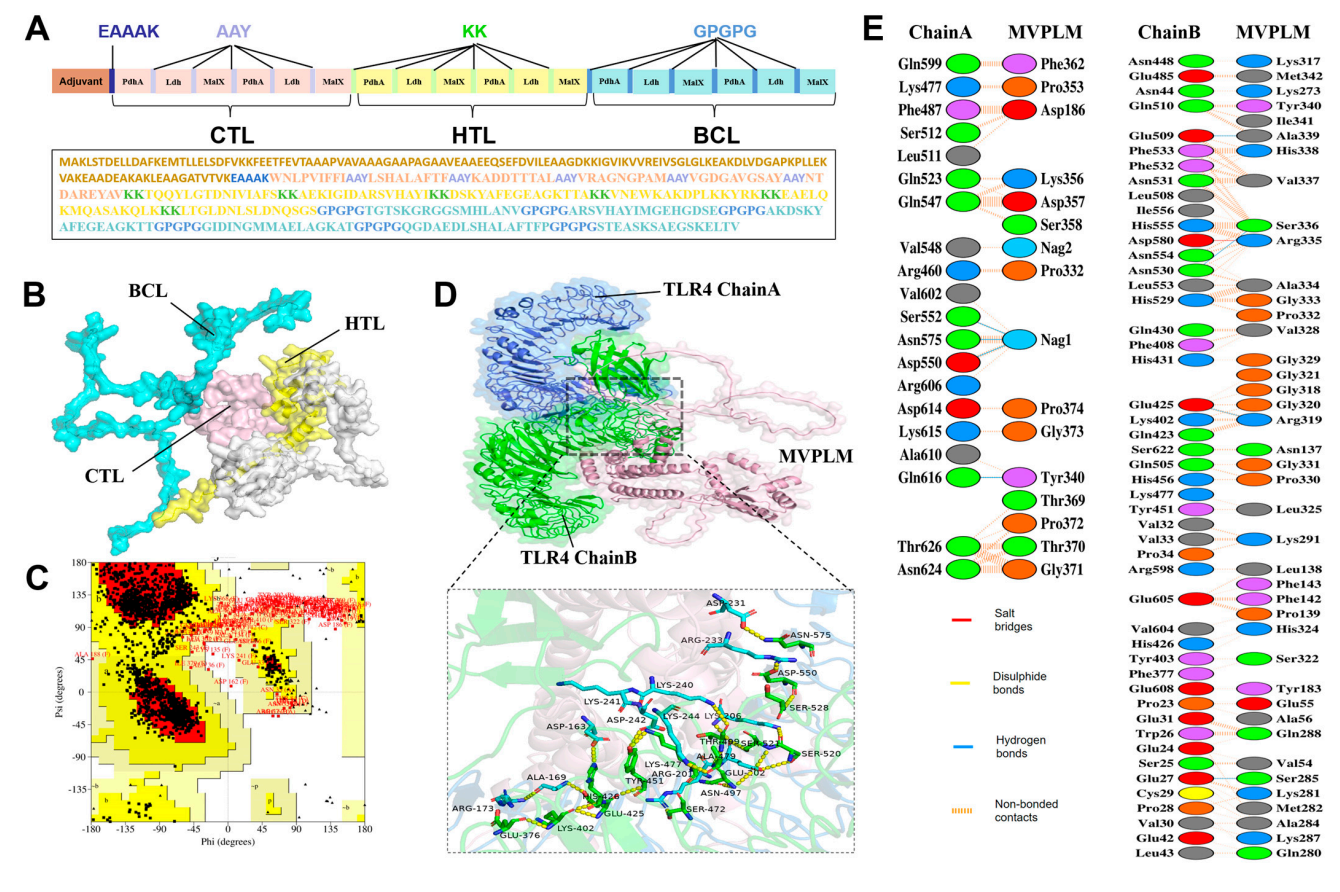
Analysis of the multi-epitope MVPLM construct and its molecular docking with TLR4. (**A**) Schematic illustration of the sequence and epitope alignment of the MVPLM construct. (**B**) The anticipated three−dimensional structure of the MVPLM. The epitopes are color−coded: pink for CTL epitopes; yellow for HTL epitopes; blue for BCL epitopes; and white for adjuvants and linkers. (**C**) The Ramachandran plot of the MVPLM construct. The red zone indicates the most favorable conformations; the yellow area represents additional permissible conformations; and the pale−yellow area denotes regions with generous allowances. (**D**) Graphical representation of the docking pattern between MVPLM and TLR4. (**E**) Residues that participate in the interaction between docked MVPLM and TLR4.

Advancements in contemporary vaccinology and immunoinformatics have led to the emergence of multi-epitope vaccines as a promising strategy for the integration of various proteins into a single vaccine. Subsequently, we attempted to develop a multi-epitope vaccine (MVPLM) composed of PdhA, Ldh, and MalX immunogenic proteins. The BCL, CTL, and HTL epitopes were predicted using ABCpred and IEDB (Tables S3–S5). Based on their high antigenicity, binding affinity, non-allergenicity, and ability to induce IL-4 and IFN-γ, the high-ranked BCL, CTL, and HTL epitopes from each protein were selected separately for subsequent vaccine construction. To create chimeric peptides, a total of 6 CTL, 6 HTL, and 6 BCL epitopes were linked using AAY, KK, and GPGPG linkers, respectively (Figure 7A). The EAAAK linker was also used to connect the initial CTL epitope to the adjuvant sequence (the 50S ribosomal protein L7/L12, which is composed of 130 amino acids). The final 3D structure of the MVPLM was obtained using the I-TASSER server (Figure 7B).

Additionally, the Ramachandran plot confirmed that 77.5% of the MVPLM residues were located in the favored regions (red zone), 17.4% in the additional allowed regions (yellow zone), 3.3% in the generously allowed regions (pale-yellow zone), and 1.8% in the disallowed regions (Figure 7C). Vaccines must exhibit a significant affinity for host immune receptors (including Toll-like receptors and MHC molecules) to elicit an effective immune response. A molecular docking analysis conducted using ClusPro 2.0 demonstrated the MVPLM’s robust binding affinity for TLR4 (Figure 7D). The interactions within the MVPLM-TLR4 complex comprised hydrogen bonds between the Ser285-Glu27, Arg319-Glu425, Ser336-Asn530, Ala339-Glu509, and Tyr340-Gln616 pairs. A PDBsum server analysis revealed the existence of one salt bridge and 114 non-bonded contacts among the interacting atoms within the MVPLM-TLR4 docked complex (Figure 7E).

### 3.6. Evaluation of the Immunization Effect of the Multi-Epitope MVPLM

**Figure 8:**
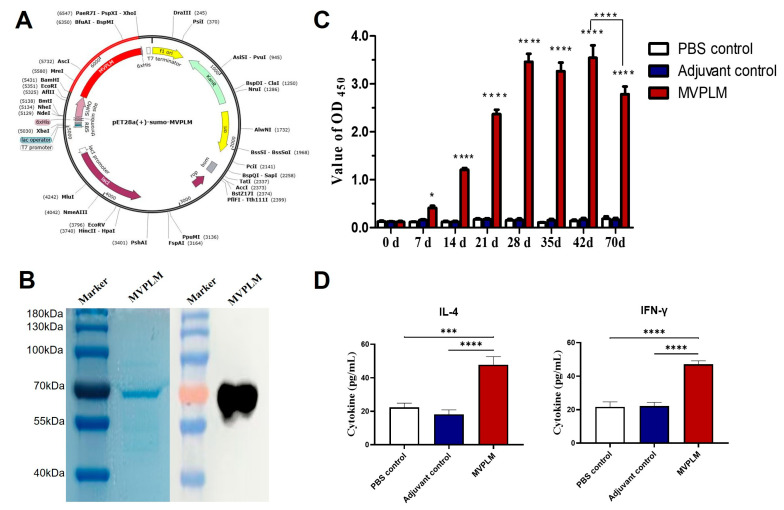
Expression, purification, and immunization of the multi-epitope MVPLM. (**A**) Expression map of pET28a (+)-sumo-MVPLM plasmid. The red area denotes the genes that encode the multi-epitope vaccine construct. (**B**) SDS-PAGE analysis of the MVPLM protein, followed by Western blot verification using the serum antibodies. (**C**) Titers of IgG antibodies to the multi-epitope MVPLM among vaccinated mice. Data are represented as mean ± SEM. All samples were analyzed using two-way analysis of variance, followed by Tukey’s multiple comparison test (* *p* < 0.05; **** *p* < 0.0001). The comparison showed extremely large effect sizes (Cohen’s d > 3.70) with high statistical power (1 − β > 0.96). (**D**) Mice serum IL-4 and IFN-γ cytokine levels, measured two weeks after the second immunization. The data represents a mean of 6 biological replicates ± SEM. All samples were analyzed using one-way analysis of variance, followed by Tukey’s multiple comparison test (*** *p* < 0.001; **** *p* < 0.0001).

We selected the BamHI and XhoI restriction sites and successfully inserted the optimized MVPLM sequence into the pET28a (+)-sumo vector, which produced a 6898 bp recombinant vector (Figure 8A). After transforming the pET28a (+)-sumo-MVPLM plasmid into *Escherichia coli* BL21 (DE3) and inducing expression, an SDS-PAGE analysis clearly indicated a target protein band at approximately 70 kDa, which is consistent with the predicted molecular weight (Figure 8B). Moreover, Western blot analysis further confirmed that the purified recombinant protein MVPLM could specifically bind to its immune serum. Thereafter, five-week-old female SPF BALB/c mice were used in the immune protection test. We collected serum samples from the mice before immunization and again at 7, 14, 21, 28, 35, 42, and 70 days after immunization to measure their antibody concentrations. The immunized group exhibited significantly higher levels of immunogen-specific IgG antibodies compared to the PBS/adjuvant controls, as well as increased serum IFN-γ and IL-4 levels (Figure 8C,D). These findings suggest that the multi-epitope MVPLM provides robust immunogenicity.

**Figure 9:**
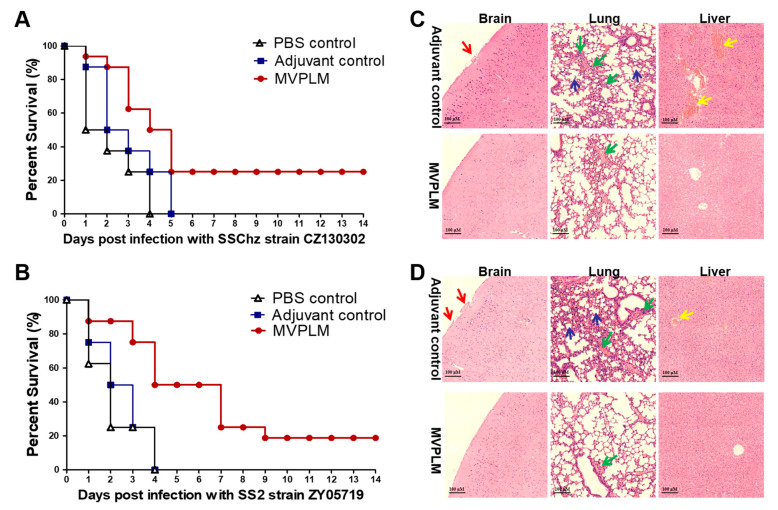
Survival rates of mice vaccinated with the multi-epitope MVPLM, and the histopathological analysis. (**A**,**B**) Immune protective effect of the recombinant protein MVPLM on mice challenged with SS CZ130302 (**A**) and ZY05719 (**B**) (*n* = 16 per group). (**C**,**D**) Histopathological analysis of brain, lung, and liver tissues from mice immunized with recombinant proteins or ISA 201 adjuvant after being challenged with CZ130302 (**C**) and ZY05719 (**D**). The red arrow indicated congestion of the meningeal vessels; the blue arrow indicated thickening of the alveolar septa and a marked reduction in alveoli; the green arrow indicated lymphocytic infiltration; and the yellow arrow indicated hepatic congestion. Scale: 100 μm.

We used the murine model to evaluate the immunization effect of the multi-epitope MVPLM. Our results indicate that the protection rate of the MVPLM against the CZ130302 strain was 25%, and 18.75% against the ZY05719 strain (Figure 9A,B). Since SS infection can cause clinical symptoms such as meningitis, pneumonia, and acute sepsis, we further evaluated the immune protective effect via histopathological analysis of brain, lung, and liver tissue from the experimental and control groups. As shown in Figure 9C,D, mild histopathological changes were observed in the brains, lungs, and livers of the mice at 12 h post-infection in the MVPLM immunized group. In the control group, the lesions were serious, and the meningeal vessels were dilated and congested. There were substantial lesions in the lungs, significant perivascular lymphocyte infiltration around the blood vessels, alveolar hemorrhage, and severe bleeding in the liver. Our results indicate that the multi-epitope MVPLM did not provide a high immune protection rate, but that it could effectively extend the survival of mice and reduce pathological damage.

## 4. Discussion

Studies monitoring the SS epidemic status have shown that the incidence of SS is very high, especially in countries with high pig densities and high raw pork consumption. Most pig farms undergo SS infections with multiple serotypes or genotypes, leading to significant public health problems and economic losses [3,40]. However, there are certain bottlenecks in the development of SS vaccines and a lack of cross-reactive vaccines that confer protection against heterologous strains of multiple serotypes. Therefore, the development of a cost-effective universal vaccine is necessary to circumvent SS infection.

Bacterial surface proteins are generally considered to be potential immunogenic proteins and candidate protective antigens for the development of vaccines. In this study, cell wall protein immunoproteomics was used to screen for candidate SS subunit vaccines. Using immunoproteomics, Wang et al. found that PaR1 secreted by T9SS is a non-serotype-specific protective protein against *Riemerella anatipestifer* [41]. Bao et al. also applied this technique to develop candidate vaccines against avian pathogenic *Escherichia coli* (APEC). They successfully identified an ideal immunogenic protein (GroEL) which effectively protected against both homologous and heterologous APEC infections [42]. In our study, we successfully identified eight immunogenic proteins (GH25, Pk, PdhA, Ldh, ExoA, Pgk, MalX, and Dnak) using immunoproteomics techniques. These proteins induce high IgG antibody titers and provide a degree of immune protection against the SS hypervirulent strain CZ130302 in the mouse infection assay.

GH25 belongs to the glycosyl hydrolase 25 family, and is an important cell adhesive in *Streptococcus pneumoniae* that plays an important role in host nasopharyngeal epithelial cell invasion [43,44,45]. This study identified a novel GH25 protein that contributes to SS virulence and may confer protective immunity. Pk protein serves as an important cell surface protein in *Streptococcus gordonii*. Additionally, it acts as a key immunogen in *Madurella mycetomatis* and is essential for the growth of *Mycobacterium tuberculosis* [46,47]. Wu et al. have confirmed that Pk is a cell wall protein in SS9 [48]. However, its specific function in SS has not been thoroughly studied. PdhA catalyzes the fermentation of pyruvate to acetyl-CoA in *Streptococcus mutans*, thereby enhancing the bacteria’s resistance to acidic and basic stresses [49,50]. Our sequence alignment and distribution results indicate that PdhA is conserved in SS, and its detection rate in SS2 is 100%. Ldh plays an important role in bacterial growth. For example, *Staphylococcus aureus* produces Ldh under nitric oxide stress, which helps maintain redox balance during nitrosative stress [51]. Zhou et al. confirmed that Ldh is an important immunogenic protein in SS and that it confers immune protection in mice [52]. This finding is consistent with our research results. Through immunoproteomics, Karlsen et al. confirmed that ExoA is an important immunogenic protein in *Salmonella* [53]. Pgk is an essential surface protein in *Streptococcus*, which could serve as a candidate subunit vaccine antigen. When combined with the whole-cell inactivated *Streptococcus agalactiae* vaccine, it effectively stimulates tilapia to produce higher levels of IL-1β, TNF-α, and antibody titers as compared to controls [54,55]. MalX is a streptococcal virulence factor that participates in carbohydrate metabolism. The oropharyngeal colonization of *Streptococcus pyogenes* is enhanced by its ability to use MalX molecules to specially utilize dietary starch [56]. Moffitt et al. further demonstrated that intranasal inoculation with SP2108 protein (which is homologous to MalX) effectively inhibits the nasopharyngeal colonization of *Streptococcus pneumoniae* in a murine model [57,58]. In our study, MalX was detected in 100% of 92 different SS strains and demonstrated good immunogenicity, indicating its potential as a candidate subunit vaccine. The heat shock protein Dnak is involved in heat stress and biofilm formation in SS [59,60]. The co-expression of DnaK and Sia10 (which form chimeric antigens) could effectively protect fish against *Edwardsiella tarda* and *Streptococcus iniae* infection [61].

Since SS has multiple serotypes and ST types, providing cross-immunity protection remains a key research priority in vaccine development. Wang et al. evaluated a novel multi-component vaccine containing three SS proteins—MRP, glyceraldehyde-3-phosphate dehydrogenase (GAPDH), and dihydrolipoamide dehydrogenase (DLD)—which provided effective protection against SS2, SS7, and SS9 infections in a zebrafish model [16]. Weisse et al. also investigated a multi-component recombinant vaccine against SS infection, which included six recombinant proteins, namely, SSU0934, SSU1869, SSU0757, SSU1950, SSU1664, and SSU0187 [15]. In our study, we screened a multi-component subunit vaccine composed of PdhA, Ldh, and MalX proteins that provided a high level of protection (75%) against SSChz CZ130302 and moderate protection (62.5%) against SS2 ZY05719 in the mouse model.

The use of multiple conserved antigenic epitopes connected by linkers has led to the development of vaccines that confer cross-protection. This has been established as a reliable and safe strategy in several bacteria, such as *Streptococcus pneumoniae* and *Staphylococcus aureus* [62,63]. Recently, researchers have also constructed several universal multi-epitope candidate vaccines against SS infection through the use of immunoinformatics. Zhang et al. designed a universal multi-epitope SS vaccine by integrating epitopes derived from three conserved immunogens (6PGD, HP0197, and PepO) identified using immunoinformatics [64]. Liang et al. developed a multi-epitope vaccine (MVSS) utilizing ten candidate epitopes derived from six proteins, which effectively mitigated lethal infection caused by SS [27]. In this study, we developed a multi-epitope subunit vaccine (MVPLM) by combining epitopes from three highly immunogenic proteins (PdhA, Ldh, and MalX) using in silico predictive methods. Although our MVPLM did not offer a high protection rate against SS in the murine model, it significantly increased serum cytokine and antibody levels, thereby mitigating pathological damage.

Although the findings of this study provide valuable insights into the multi-component and multi-epitope vaccines against SS, a key limitation is the use of a mouse model. Because physiological and immunological differences between species may affect the applicability of the findings, caution should be exercised when extrapolating these results to swine. Building upon these promising murine results, our subsequent research primarily focuses on further evaluating the protective efficacy of the PdhA-Ldh-MalX multi-component subunit vaccine in a swine model, which represents a more clinically relevant and natural host for SS infection.

## 5. Conclusions

In our research, eight immunogenic proteins were identified using immunoproteomics. The sequence alignment and gene distribution results show that PdhA, Ldh, Pgk, MalX, and Dnak were highly conserved in different SS strains. Based on the conservation and immune protection conferred by the candidate proteins screened in this study (PdhA, Ldh, and MalX), the multi-component and multi-epitope vaccines composed of these proteins effectively immunized mice against lethal infection of SS2 ZY05719 and Chz CZ130302 strains. This study provides a new avenue for the development of SS vaccines that confer multi-serotype cross-protection.

## Figures and Tables

**Table 1:** Protein spots identified by MALDI-TOF-MS.

Spot Munber	Identified Protein			Peptides Matched	Protein Score	Score C.I.%	Intensity Matched
	Mascot Results	Annotation	MW(Da)/pI				
1	gi|353533998	Family 25 glycosyl hydrolase (GH25)	39,164.4/5.52	15	347	100	68.239
2	gi|353533998	Family 25 glycosyl hydrolase (GH25)	39,164.4/5.52	12	207	100	43.623
3	gi|353533998	Family 25 glycosyl hydrolase (GH25)	39,164.4/5.52	14	73	99.539	16.845
4	gi|353533539	Pyruvate kinase (Pk)	56,311.1/5.19	28	226	100	58.357
5	gi|353533539	Pyruvate kinase (Pk)	56,311.1/5.19	20	209	99.832	57.876
6	gi|353737021	Acetoin dehydrogena-se E1 component alpha subunit (PdhA)	35,447.7/4.91	9	59	95.325	54.352
9	gi|166223157	L-lactate dehydrogenase (Ldh)	35,400.3/5.05	8	56	97.957	46.845
10	gi|556049758	Exodeoxyribonuclease (ExoA)	31,017.7/5.52	11	63	95.705	18.344
13	gi|353737491	Phosphoglycerate kinase (Pgk)	42,016.1/4.86	25	746	99.786	79.568
14	gi|353737491	Phosphoglycerate kinase (Pgk)	42,016.1/4.86	23	346	99.786	79.568
15	gi|353532515	ABC transporter subs-trate binding protein maltose (MalX)	44,358.8/4.53	17	196	100	20.541
16	gi|661304267	Molecular chaperone DnaK (Dnak)	64,746.3/4.62	28	835	100	85.457

**Table 2:** Protective efficacies of eight recombinant proteins in the mouse infection assay.

Group	No. of Surviving/Total Infected Mice	Survival Percent
GH25	6/16	37.5%
Pk	6/16	37.5%
PdhA	8/16	50%
ExoA	2/16	12.5%
Ldh	8/16	50%
Pgk	6/16	37.5%
MalX	6/16	37.5%
Dnak	4/16	25%
Adjuvant control	0/16	0%
PBS control	0/16	0%

## Data Availability

Data available on request due to privacy restrictions.

## Supplementary Materials

The following supporting information can be downloaded at: https://www.mdpi.com/article/10.3390/vaccines13101020/s1, Table S1: Bacterial strains, plasmids and primers used in this study. Table S2: Gene distribution of 92 *Streptococcus suis* clinical isolates. Table S3: The identification and evaluation of BCL epitopes. Table S4: The identification and evaluation of CTL epitopes. Table S5: The identification and evaluation of HTL epitopes.
