# Supplementary material for: Immunoproteomic Screening of Candidate Antigens for the Preliminary Development of a Novel Multi-Component and Multi-Epitope Vaccine Against Streptococcus suis Infection
Source: Vaccines (Basel). 2025 Sep 30;13(10):1020. doi: 10.3390/vaccines13101020 (PMC12568063; doi:10.3390/vaccines13101020)
Supplement: Supplementary file 1 [file vaccines-13-01020-s001.zip › Table S1.pdf]

Table S1. Bacterial strains, plasmids and primers used in this study.

Strain, plasmid or primer	Description[a] or sequences (5′–3′)[b]	Sources or function
**Bacterial strains**		
CZ130302	A novel variant serotype Chz of SS which caused acute meningitis in piglets	Collected in our lab
ZY05719	Virulent strain of the SS2	Collected in our lab
S191	Virulent strain of the SS3	Collected in our lab
SH04815	Virulent strain of the SS7	Collected in our lab
SH896	Virulent strain of the SS9	Collected in our lab
DH5α	Host for maintaining the recombinant plasmids	Invitrogen
BL21(DE3)	Host for expressing the recombinant proteins	Invitrogen
**plasmids**		
pET28a (+)	Expression vector, Kan^+	Invitrogen
pET28a (+)-sumo	Expression vector, Kan^+	Invitrogen
**Primers for construction of expression vectors**		
GH25-L	CGgaattc AAATTGGATAATATTAAAGGT	Primers for construction of
GH25-R	CCGctcgag TTATTTAGCTGAGTAGAAAGC	pET-28a-GH25
Pk-L	CGgaattc ACCCTTGGTCCTGCGGTAG	Primers for construction of
Pk-R	CCGctcgag TTATGTACGAACACGCATAGT	pET-28a-Pk
PdhA-L	CGgaattc ATCTCAAAAGAACAACACTTGG	Primers for construction of
PdhA-R	CCGctcgag CTAATCTACAAACACGTCTTCG	pET-28a-PdhA
ExoA-L	CGgaattcTCTTGGAACATTGATTCTCTT	Primers for construction of
ExoA-R	CCGctcgag TTATAGCTCAATTTCCATCAC	pET-28a-ExoA
Ldh-L	CGgaattc GTAATCCTTGTCGGTGACGGT	Primers for construction of
Ldh-R	CCGctcgag TTAGTTTTTACACCAGCTGC	pET-28a-Ldh
Pgk-L	CGgaattcATGGCAAAATTGACTGTTAAAG	Primers for construction of
Pgk-R	CCGctcgag TTACTTTTCAGTCAAAGCTGC	pET-28a-Pgk
MalX-L	CGgaattcGAAGGTAGCAAAGAATTGACT	Primers for construction of
MalX-R	CCGctcgag CTAGTTGCTGTGTTTTTGAGC	pET-28a-MalX
Dnak-L	CGggatccGGTACAACAAACTCAGCAGTT	Primers for construction of
Dnak-R	CCctcgagTTACTCCGTAAACTCGCCGTC	pET-28a-Dnak
**Primers for detection in SS**		
GH25-U	TGACACGCAAGCTGTTACTGAT	Primers for detection of
GH25-D	TTGCGTCTGTTGTGTAAAGG	*gH25* gene in SS
Pk-U	TACCGAAGGTGCAAACGTATTC	Primers for detection of
Pk-D	AACCTTGTTCCAAACCGAAAC	*pk* gene in SS
PdhA-U	CTTCTCAGGTGACTCAGCGACA	Primers for detection of

PdhA-D	TTCTGCCACTTGTGCTTCGAT	*pdhA* gene in SS
ExoA-U	CAGAGTCTCCTCGCGCCCTATT	Primers for detection of
ExoA-D	AATCCTGGTGACTGGCGGTTGC	*exoA* gene in SS
Ldh-U	GTGCCGTAGGTTCTGCTTATGC	Primers for detection of
Ldh-D	CTCATCAATATCGCGGTTAT	*ldh* gene in SS
Pgk-U	TTCATCGCAGGTGCTACTCGT	Primers for detection of
Pgk-D	ACTGGCAAGATCAATTTACCGT	*pgk* gene in SS
MalX-U	GATGGCACCATACGACCGTGTA	Primers for detection of
MalX-D	TTTGATGGCACCTTCATTAGCA	*malX* gene in SS
Dnak-U	GTATCCCAGCCGTTGTAG	Primers for detection of
Dnak-D	TCAAGTTTGGCTTTCATGTCGT	*dnak* gene in SS

[a] Kan$^+$, kanamycin resistance cassette.

[b] Lowercase nucleotides denote restriction enzyme sites.
