# Supplementary material for: Immunoproteomic Screening of Candidate Antigens for the Preliminary Development of a Novel Multi-Component and Multi-Epitope Vaccine Against Streptococcus suis Infection
Source: Vaccines (Basel). 2025 Sep 30;13(10):1020. doi: 10.3390/vaccines13101020 (PMC12568063; doi:10.3390/vaccines13101020)
Supplement: Supplementary file 1 [file vaccines-13-01020-s001.zip › Table S2.pdf]

**Table S2. Gene distribution of 92 *Streptococcus suis* clinical isolates.**

| Strains | *gH25* | *pk* | *pdhA* | *exoA* | *ldh* | *pgk* | *malX* | *dnak* |
|---|---|---|---|---|---|---|---|---|
| ***Streptococcus suis* serotype 2** | | | | | | | | |
| HA9801 | - | + | + | + | + | + | + | + |
| SS2-1-1 | - | + | + | + | + | + | + | + |
| ZY05719 | - | + | + | + | + | + | + | + |
| GH05458 | - | + | + | + | + | + | + | + |
| ZG05464 | - | + | + | + | + | + | + | + |
| JDZ05802-1 | + | + | + | + | + | + | + | + |
| CHZ05806 | - | + | + | + | + | + | + | + |
| BB070118 | - | + | + | + | + | + | + | + |
| SS07-01 | - | + | + | + | + | + | + | + |
| 07-ss-NJ-01 | - | + | + | + | + | + | + | + |
| 07-ss-RG-02 | - | + | + | + | + | + | + | + |
| NH4 | - | + | + | + | + | + | + | + |
| hb1002 | - | + | + | + | + | + | + | + |
| hb1007 | - | + | + | + | + | + | + | + |
| hb1012 | - | + | + | + | + | + | + | + |
| SS2-TY | - | + | + | + | + | - | + | + |
| YY060816 | - | + | + | + | + | + | + | + |
| SC070807 | - | + | + | + | + | + | + | + |
| 6035 | - | + | + | + | + | + | + | + |
| HN07137 | - | + | + | + | + | + | + | + |
| HN08324 | - | + | + | + | + | + | + | + |
| JX071102 | - | + | + | + | + | + | + | + |
| JX071204 | - | + | + | + | + | + | + | + |
| TZ080501 | - | + | + | + | + | + | + | + |
| LP081102 | - | + | + | + | + | + | + | + |
| LP09106 | - | + | + | + | + | + | + | + |
| ZJHN090702 | + | + | + | + | + | + | + | + |
| ZJHN090705 | + | + | + | + | + | + | + | + |
| ZJJX0908001 | + | + | + | + | + | + | + | + |
| ZJJX0908004 | + | + | + | + | + | + | + | + |
| HN1004001 | + | + | + | + | + | + | + | + |

| | | | | | | | | |
|---|---|---|---|---|---|---|---|---|
| JX1105 | - | + | + | + | + | + | + | + |
| ZJ92091101 | - | + | + | + | + | + | + | + |
| HN0104001 | - | + | + | + | + | + | + | + |
| w09001 | - | + | + | + | + | + | + | + |
| 08JX1202 | + | + | + | + | + | + | + | + |
| XS06026 | + | + | + | + | + | + | + | + |
| JX071103 | + | + | + | + | + | + | + | + |
| SS2Y | + | + | + | + | + | + | + | + |
| SS259A | + | + | + | + | + | + | + | + |
| SS2159E | + | + | + | - | + | + | + | + |
| SS2HA | - | + | + | - | + | + | + | + |
| SS2-191 | - | + | + | + | + | + | + | + |
| SS2-97A | - | + | + | + | + | + | + | + |
| SS2-259 | - | + | + | + | + | + | + | + |
| 129-2 | - | + | + | - | + | + | + | + |
| ZJNB115 | - | + | + | - | + | + | + | + |
| 700795 | - | + | + | - | + | + | + | + |
| ***Streptococcus suis* serotype 3** | | | | | | | | |
| Hb1001 | - | - | - | + | + | + | + | + |
| yn19 | - | + | + | + | + | + | + | + |
| yn20 | + | + | + | + | + | + | + | + |
| yn21 | - | - | - | + | + | + | + | + |
| SS111-3-1 | - | - | + | + | + | + | + | + |
| S191 | + | + | + | + | + | + | + | + |
| ***Streptococcus suis* serotype 7** | | | | | | | | |
| yn4 | - | + | + | + | + | + | + | + |
| yn6 | - | - | - | + | + | + | + | + |
| yn8 | + | + | + | + | + | + | + | + |
| SH59 | - | + | + | + | + | - | + | + |
| SH04815 | + | + | + | + | + | + | + | + |
| 0911-037m-x | + | + | + | + | + | + | + | + |
| 0911-065m-1 | - | + | + | + | + | + | + | + |
| SH04805 | + | + | + | + | + | + | + | + |
| 0911-065m-2 | - | + | + | + | + | + | + | + |

| | | | | | | | | |
|---|---|---|---|---|---|---|---|---|
| XTR2 | - | + | + | + | + | + | + | + |
| ***Streptococcus suis* serotype 9** | | | | | | | | |
| SH65 | - | - | + | - | + | + | + | + |
| yn1 | + | + | + | + | + | + | + | + |
| yn2 | + | + | + | - | + | + | + | + |
| yn3 | + | - | + | - | + | + | + | + |
| yn7 | + | + | + | + | + | + | + | + |
| yn14 | + | + | + | + | + | + | + | + |
| SH040917 (40) | + | + | + | + | + | + | + | + |
| JX041226 (55) | + | + | + | + | + | + | + | + |
| SH06 | - | + | + | + | + | + | + | + |
| SH896 | + | + | + | + | + | + | + | + |
| NJ-1 | + | + | + | + | + | + | + | + |
| NJ-2 | - | + | + | + | + | + | + | + |
| NJ-3 | - | + | + | + | + | + | + | + |
| NJ-4 | + | + | + | + | + | + | + | + |
| NJ-5 | + | + | + | + | + | + | + | + |
| NJ-6 | + | + | + | + | + | + | + | + |
| 7083 | + | - | + | + | + | + | + | + |
| SH89 | + | + | + | + | + | + | + | + |
| SH26 | - | + | + | + | + | + | + | + |
| SS27 | - | + | - | + | + | + | + | + |
| SS171 | - | + | + | + | + | + | + | + |
| hb1019 | - | + | + | + | + | + | + | + |
| 131201 | - | + | + | + | + | + | + | + |
| zg1004 | - | + | + | + | + | + | + | + |
| ***Streptococcus suis* serotype Chz** | | | | | | | | |
| CZ130302 | + | + | + | + | + | + | + | + |
| CZ110902 | + | + | + | + | + | + | + | + |
| HN136 | - | + | + | + | + | + | + | + |
| AH681 | - | + | + | + | + | + | + | + |
| | 34/92 | 85/92 | 88/92 | 84/92 | 92/92 | 90/92 | 92/92 | 92/92 |
