# Supplementary material for: Immunoproteomic Screening of Candidate Antigens for the Preliminary Development of a Novel Multi-Component and Multi-Epitope Vaccine Against Streptococcus suis Infection
Source: Vaccines (Basel). 2025 Sep 30;13(10):1020. doi: 10.3390/vaccines13101020 (PMC12568063; doi:10.3390/vaccines13101020)
Supplement: Supplementary file 1 [file vaccines-13-01020-s001.zip › Table S3.pdf]

Table S3. The identification and evaluation of BCL epitopes.

Protein	Peptide	Position	Score	Antigenicity
PdhA	TGTSKGRGGSMHLANV	91-106	0.89	1.7419
	VKFAQESPDPDISVAY	301-316	0.83	0.8588
	LDAIEAQVAEQVEASV	286-301	0.81	0.9408
	GIDINGMMAELAGKAT	76-91	0.80	1.3708
	HGHVIAKGIDINGMMA	69-84	0.77	0.9805
	AGNGPAMVEVESYRWF	228-243	0.77	0.8836
Ldh	GETRLELVEKNLRINQ	89-104	0.85	0.9711
	ARSVHAYIMGEHGDSE	168-183	0.83	1.3476
	SGTSLDSARFRQALAE	147-162	0.83	0.9006
	QGDAEDLSHALAFTFP	46-61	0.77	0.9711
MalX	ADDSKADDTTTALVTN	117-132	0.83	1.1343
	AKDSKYAFEGEAGKTT	169-184	0.81	1.3687
	GSLGSEGQLSELTLAD	103-118	0.81	0.9475
	DVKADFEKENGVTVTV	55-70	0.80	1.2250
	TNEVPANTDAREYAVS	337-352	0.79	0.9129
	STEASKSAEGSKELTV	28-43	0.79	1.7635
