# Supplementary material for: Immunoproteomic Screening of Candidate Antigens for the Preliminary Development of a Novel Multi-Component and Multi-Epitope Vaccine Against Streptococcus suis Infection
Source: Vaccines (Basel). 2025 Sep 30;13(10):1020. doi: 10.3390/vaccines13101020 (PMC12568063; doi:10.3390/vaccines13101020)
Supplement: Supplementary file 1 [file vaccines-13-01020-s001.zip › Table S4.pdf]

Table S4. The identification and evaluation of CTL epitopes.

Protein	Peptide	Position	Allele	Antigenicity	Immunogenicity
PdhA	GTDNIVIAF	135-143	SLA-1*0101	1.1517	0.30433
	MNLAAVWNL	158-166	SLA-6*0105	0.9306	0.29589
	WNLPVIFFI	164-172	SLA-6*0105	2.1423	0.31792
	SVGEEAAAV	42-50	SLA-6*0105	1.1802	0.30196
	VRAGNGPAM	226-234	SLA-3*0602	1.7010	0.08590
	RGHGHVIAK	67-75	SLA-3*0602	1.0280	0.25014
	ESPDPDISV	306-314	SLA-2*0502	1.4455	0.04446
Ldh	ANPEIAAGV	317-325	SLA-1*0101	1.0388	0.3164
	IMGEHGDSEF	175-184	SLA-1*0801	0.7883	0.11601
	LSHALAFTF	52-60	SLA-2*0101	1.0430	0.19738
	VGDGAVGSAY	13-22	SLA-1*0701	1.6517	0.03473
	IPLSEAELQ	291-299	SLA-1*0701	0.7070	0.04228
MalX	KADDTTTAL	121-129	SLA-1*0101	1.2078	0.15948
	KNDELTTAV	353-361	SLA-1*0101	0.8807	0.18931
	VPANTDAREY	340-349	SLA-1*0701	0.9937	0.2046
	NTDAREYAV	343-351	SLA-2*0502	1.3156	0.21096
