# Supplementary material for: Immunoproteomic Screening of Candidate Antigens for the Preliminary Development of a Novel Multi-Component and Multi-Epitope Vaccine Against Streptococcus suis Infection
Source: Vaccines (Basel). 2025 Sep 30;13(10):1020. doi: 10.3390/vaccines13101020 (PMC12568063; doi:10.3390/vaccines13101020)
Supplement: Supplementary file 1 [file vaccines-13-01020-s001.zip › Table S5.pdf]

Table S5. The identification and evaluation of HTL epitopes.

Protein	Peptide	Position	Antigenicity	IL4
PdhA	TQQYLGTDNIVIAFS	130-144	0.7534	inducer
	VNEWKAKDPLKKYRK	259-273	0.5920	inducer
	GGGYALAVGAALTQQ	118-132	0.5742	inducer
	ENKIATDEELDAIEA	277-291	0.6147	non-inducer
Ldh	SARFRQALAEKIGID	153-167	0.4306	inducer
	AEKIGIDARSVHAYI	161-175	1.9478	inducer
	GFNGIFLVAANPVDV	114-128	0.9102	non-inducer
	EAELQKMQASAKQLK	295-309	0.4419	Inducer
MalX	DSKYAFEGEAGKTTA	171-185	1.6641	inducer
	LTGLDNLSLDNQSGS	76-90	0.7914	inducer
	AREYAVSKNDELTTA	346-360	0.4624	inducer
